# Enteric neurons from Parkinson’s disease patients display *ex vivo* aberrations in mitochondrial structure

**DOI:** 10.1038/srep33117

**Published:** 2016-09-14

**Authors:** A. S. Baumuratov, P. M. A. Antony, M. Ostaszewski, F. He, L. Salamanca, L. Antunes, J. Weber, L. Longhino, P. Derkinderen, W. J. H. Koopman, N. J. Diederich

**Affiliations:** 1 Luxembourg Centre for Systems Biomedicine, University of Luxembourg, Campus Belval, 7, avenue des Hauts-Fourneaux, L-4362 Esch-sur-Alzette, Luxembourg; 2 Department of Infection and Immunity, Luxembourg Institute of Health, 29, rue Henri Koch, L-4354 Esch-sur-Alzette, Luxembourg; 3 Integrated Biobank of Luxembourg, 6, rue Nicolas Ernest Barblé, L-1210, Luxembourg; 4 Department of Gastroenterology, Centre Hospitalier de Luxembourg, 4, rue Barblé, L-1210, Luxembourg; 5 Department of Neurosciences, Centre Hospitalier de Luxembourg, 4, rue Barblé, L-1210, Luxembourg; 6 Department of Neurology, CHU Nantes, F-44093, France; 7 Department of Biochemistry (286), Radboud Institute for Molecular Life Sciences (RIMLS), Nijmegen Center for Mitochondrial Medicine (RCMM), Radboudumc, Nijmegen, The Netherlands

## Abstract

Based on autopsy material mitochondrial dysfunction has been proposed being part of the pathophysiological cascade of Parkinson’s disease (PD). However, in living patients, evidence for such dysfunction is scarce. As the disease presumably starts at the enteric level, we studied ganglionic and mitochondrial morphometrics of enteric neurons. We compared 65 ganglia from 11 PD patients without intestinal symptoms and 41 ganglia from 4 age-matched control subjects. We found that colon ganglia from PD patients had smaller volume, contained significantly more mitochondria per ganglion volume, and displayed a higher total mitochondrial mass relative to controls. This suggests involvement of mitochondrial dysfunction in PD at the enteric level. Moreover, in PD patients the mean mitochondrial volume declined in parallel with motor performance. Ganglionic shrinking was evident in the right but not in the left colon. In contrast, mitochondrial changes prevailed in the left colon suggesting that a compensatory increase in mitochondrial mass might counterbalance mitochondrial dysfunction in the left colon but not in the right colon. Reduction in ganglia volume and combined mitochondrial morphometrics had both predictive power to discriminate between PD patients and control subjects, suggesting that both parameters could be used for early discrimination between PD patients and healthy individuals.

Mitochondria are among the prime suppliers of energy (ATP) in virtually all living cells^1^,^2^ and empirical evidence suggests that their dysfunction is involved in the pathomechanism of Parkinson’s disease (PD)^3^,^4^,^5^. Despite considerable progress, the potential role of mitochondrial (dys)function during PD development remains poorly understood. One of the main reasons for this lack of insight is the fact that there is no direct *in vivo* access to nigrostriatal dopaminergic neurons, the loss of which is one of the main pathological features of PD^6^. The latter study hypothesized the gastrointestinal tract may be the starting point of the disease and thus enteric neurons may reliably reflect fundamental disease characteristics. Indeed, both Lewy neurites and Lewy bodies (LB), abnormal protein aggregates that develop in nerve cells during PD, have been demonstrated in the submucosal colon nerve layer of patients with early PD^7^,^8^,^9^,^10^,^11^. This suggests that pathological aberrations observed in enteric neurons might represent an early stage of PD manifestation^7^,^8^. Furthermore, animal studies of enteric neurons suggest a direct involvement of mitochondrial dysfunction in PD. It was suggested that inhibitory enteric neurons are particularly vulnerable to mitochondrial dysfunction induced by Parkinsonian neurotoxins^12^. In this sense, mice treated with a chemical inhibitor of the first mitochondrial oxidative phosphorylation (OXPHOS) complex (complex I), displayed delayed gastric emptying in a dosage-dependent manner^12^,^13^. Similarly, severe gastrointestinal dysmotility associated with degeneration of enteric neuronal and glial cells was demonstrated in a transgenic mouse model displaying impaired mitochondrial metabolism in these cells^14^. Accumulating evidence suggests that cell metabolism and mitochondrial (dys)function are linked to mitochondrial (ultra)structure and motility^1^,^15^,^16^,^17^,^18^. Changes in mitochondrial morphology have been demonstrated in fibroblasts of PD patients with LRRK2 or Parkin mutations^19^,^20^. This suggests that mitochondrial morphology changes might be similarly indicative of the ongoing disease process in idiopathic PD. Here we investigated this hypothesis by quantitatively comparing 3D mitochondrial morphology and ganglion volume between colon biopsies from PD patients without clinically apparent gastrointestinal dysfunction and age-matched control subjects. We developed an image quantification procedure allowing unbiased 3D analysis of mitochondrial morphology and ganglion volume in immunofluorescence images obtained by confocal microscopy. This revealed that ganglion volume and mitochondrial morphology were aberrant in PD patients and that these changes allowed discrimination between PD patients and healthy individuals.

## Results

The enrolled subjects were of similar age (PD patients: 70 ± 6 years; controls: 65 ± 5 years; P = 0.17). There were five male and six female patients and one male and three female controls. In patients the average disease duration at the time of colon biopsy was 5.4 ± 5.7 years. The average Unified Parkinson’s Disease Rating Scale (UPDRS) motor score describing disease progression equaled 10.7 ± 8.1. The mean levodopa equivalent dosage was 630.3 ± 483 mg^21^. Following random selection of the biopsies, we analyzed 65 biopsies from PD patients and 41 biopsies from healthy subjects in detail.

### Image analysis algorithm and parameters

**Figure 1:**
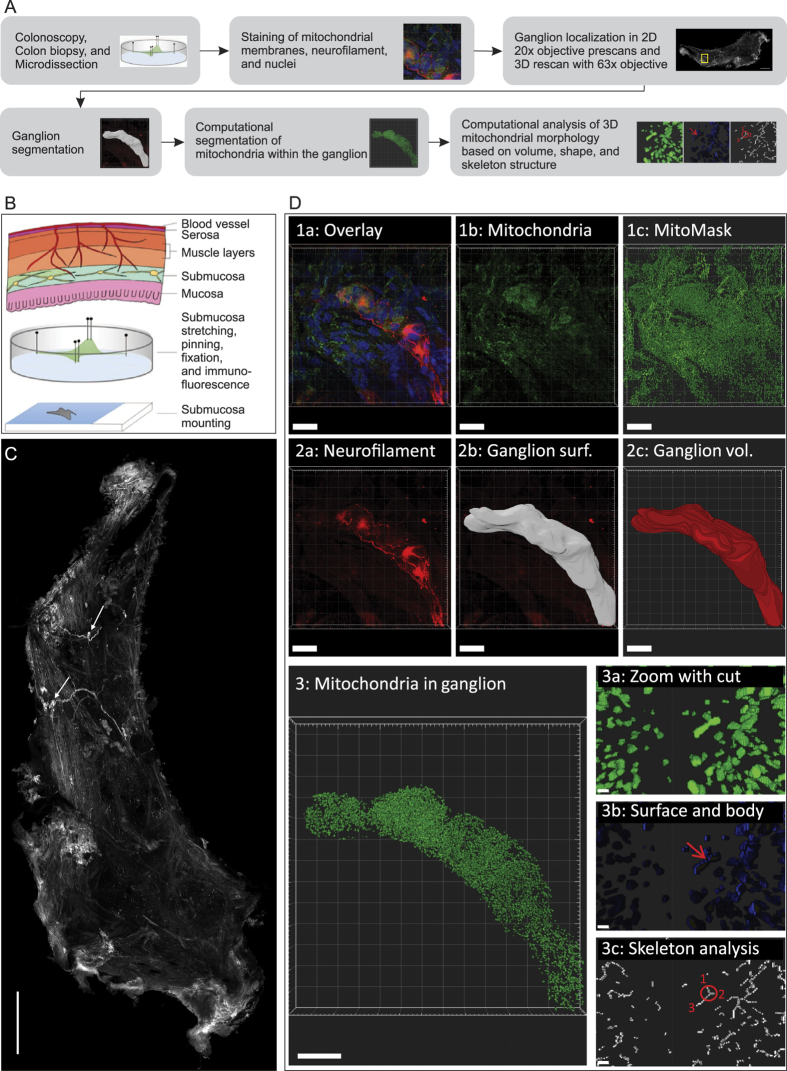
Workflow and image analysis procedure: The summarized workflow from colonoscopy to the analysis of 3D mitochondrial morphology is shown in (**A**). Schematic location of the submucosa layer and colon biopsy processing steps (**B**). Stretched biopsy represented as mosaic composed of 77 stitched images from the neurofilament channel (**C**). The arrows point to individual ganglia. Scale bar = 500 μm. Image analysis steps (**D**): Mitochondrial volumes were defined via computational image analysis (D1abc). Ganglion masks were defined via manual surface selection from the neurofilament channel (D2ab) and transformed to volume information (D2c). All mitochondria not in contact with ganglia were removed (D3). Morphological analysis on segmented mitochondrial volumes was based on the distinction between mitochondrial surface and body (D3b) and on the analysis of mitochondrial branching (D3c). Branching analysis was based on the count of mitochondrial nodes. In the example highlighted in red, not only the circled branchpoint but also the mitochondrial endpoints labeled 1, 2, and 3 are counted as nodes. Node degrees, as defined by the adjacency matrix proposed by Kerschnitzki *et al*.^24^, do not only count branches connected to a node (3 in this example), but the cumulated count of skeleton pixels in these branches. Scale bars for D1abc, D2abc, D3: 20 μm. Scale bars for D3abc: 0,5 μm.

**Figure 2:**
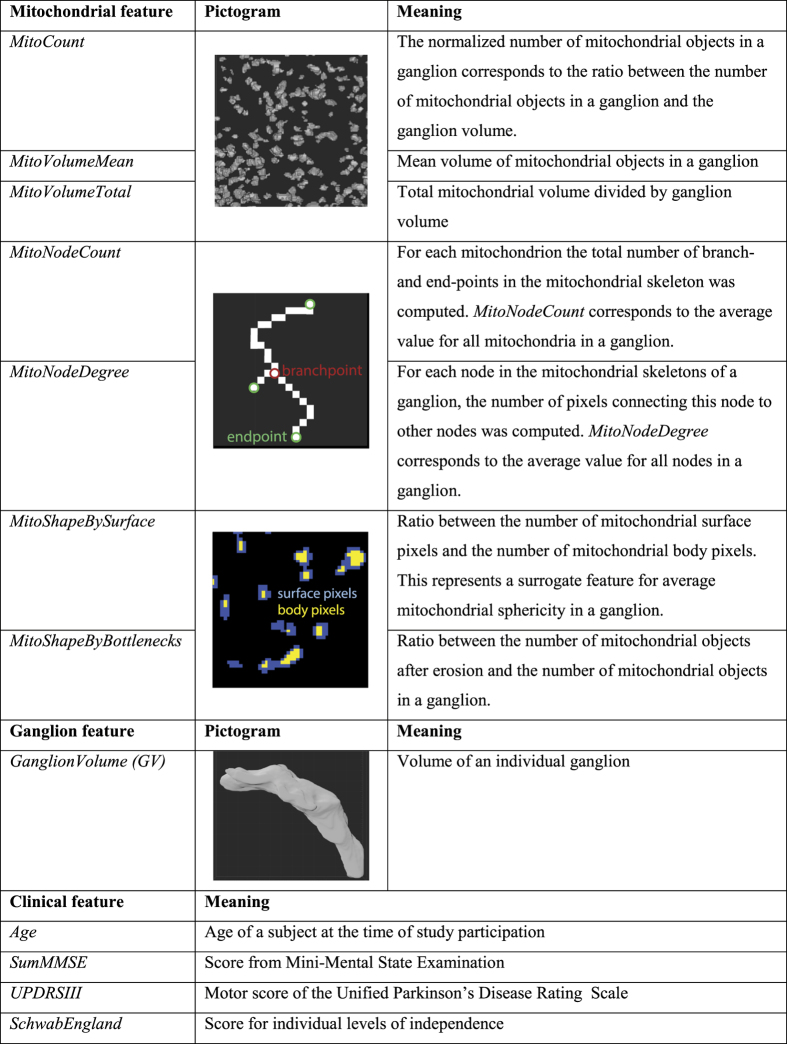
Morphometric and clinical features used in this work.

The biopsies were processed as illustrated in Fig. 1. For automated segmentation of mitochondria within a ganglion, deconvolved Alexa-488 channel images were adjusted to 16 bit by multiplying each pixel value with the ratio between the theoretical maximum of a 16 bit pixel, namely 2^16^ − 1, and the maximum pixel value of the 12 bit image to be adjusted. Mitochondrial pixels were defined using two rules: first, local thresholds had to confirm that local signals obtained from a 5 pixel sized Gaussian filter with a standard deviation of 2 were at least 25% brighter than the surrounding background, as defined by a 10 × 10 average filter. Second, foreground signals in non-uniformly corrected image stacks had to be confirmed via global thresholds according to the Otsu method, in which an optimal threshold is selected by maximizing the measure of separability between foreground and background in terms of gray levels^22^. Correction of non-uniform illumination was performed by subtracting 20 × 20 average filtered images from the corresponding deconvolved images. To remove shot noise, which occurs in photon counting devices, connected components fulfilling both mitochondrial pixel recognition criteria were filtered for a volume of at least eight pixels^23^. Objects with more than 10^6^ pixels were removed from the mitochondria mask. Mitochondrial feature extraction was restricted to connected components intersecting with the ganglion volume. For segmentation of mitochondrial surface and mitochondrial body, the mitochondrial mask was eroded with a structuring element defined by a center pixel and its 6-connected neighbourhood. Skeletonization of the mitochondrial 3D mask was performed as described before^24^. Figure 1 and the Supp. Movie 1 shows the resulting mitochondrial volumes, surfaces, eroded bodies, and skeletons in 3D. In addition, we show the mitochondrial state in both healthy controls and PD patients (Supplementary Figure 1). Overall, 7 features describing mitochondrial morphology in a ganglion were analyzed Fig. 2.

### Mitochondrial morphometrics

**Figure 3:**
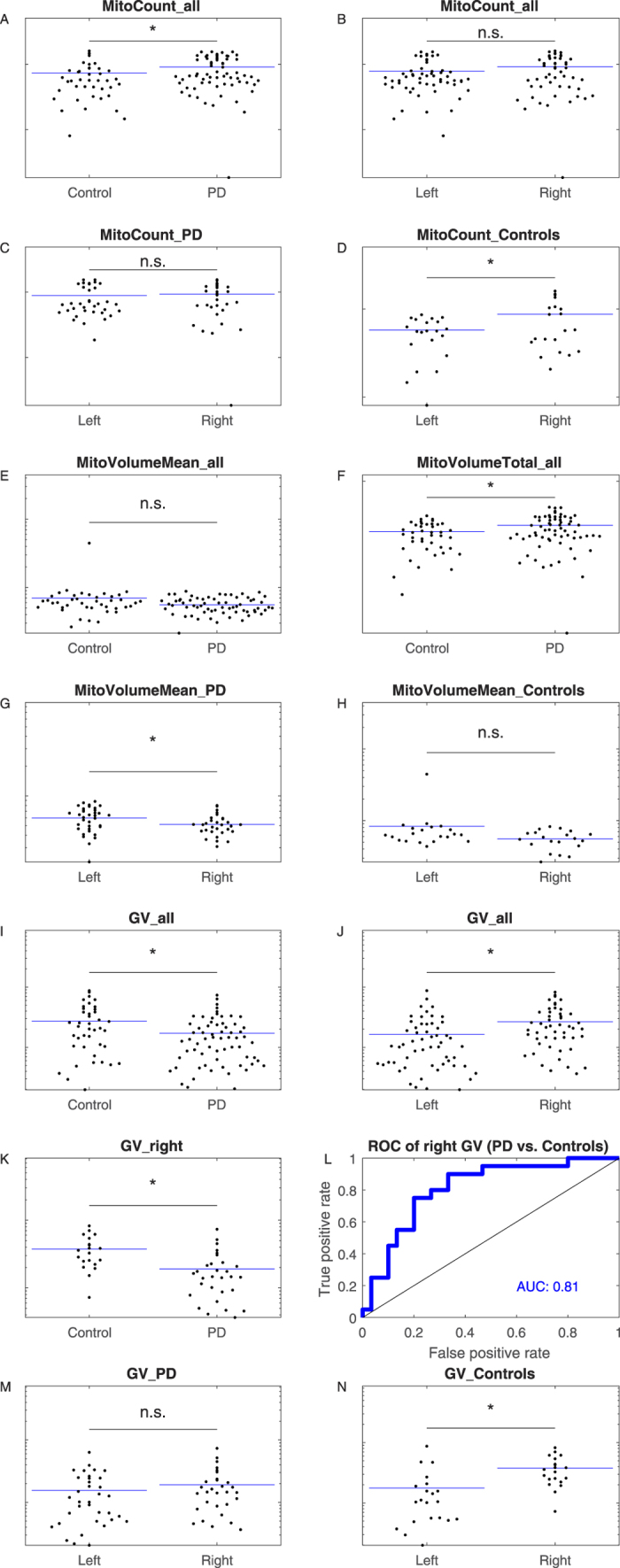
Mitochondria and ganglia morphometrics of enteric neurons from patients and controls. (**A**) Mitochondria count per volume of patients and controls (p = 0.043). (**B**) Mitochondria count per volume between left and right colon location independently of disease status (p = 0.11). (**C**) Mitochondria count per volume compared between left and right colon location from patients only (p = 0.61). (**D**) Mitochondria count per volume compared between left and right colon location from controls only (p = 0.022). (**E**) Mean mitochondria volume of patients and controls (p = 0.197). (**F**) Normalized mitochondrial mass of patients and controls (p = 0.017). (**G**) Mean mitochondria volume comparison between left and right colon location from patients only (p = 0.028). (**H**) Mean mitochondria volume comparison between left and right colon location from controls only (p = 0.076). (**I**) Ganglia volume of patients and controls (p = 0.005). (**J**) Ganglia volume comparison between left and right colon location independently of disease status (p = 0.004). (**K**) Ganglia volumes from the right colon of patients and controls (p = 0.0006). (**L**) ROC analysis. The corresponding p-value 2.3e-4 indicates the random chance that the AUC is not different from 0.5 (null hypothesis: AUC = 0.5). (**M**) Ganglion volumes of patients compared between left and right colon (p = 0.33). (**N**) Ganglion volumes of controls compared between left and right colon (p = 0.002). All features are plotted on log scales (**A**–**K**,**M**,**N**). AUC = area under the curve; PD = Parkinson’s disease; ROC = receiver operating curve.

3D immunofluorescence image stacks were acquired by confocal microscopy from fixed colon biopsies, as described above. These stacks were used to first reconstruct and subsequently quantify 3D ganglion and mitochondrial structural features. The number of mitochondrial objects per ganglion, corrected for ganglion volume (Fig. 2; *MitoCount*), was higher in PD patients than in control subjects (Fig. 3A; P = 0.043). In the whole cohort (including PD patients and controls), there was no difference in *MitoCount* between the right and left colon (Fig. 3B; P = 0.11). Also in PD patients *MitoCount* was not significantly different between the right and left colon (Fig. 3C; P = 0.61). In control individuals, in contrast, *MitoCount* was higher in the right colon than in the left colon (Fig. 3D; P = 0.022). Total mitochondrial volume normalized to ganglion volume (Fig. 2; *MitoVolumeTotal*) is a measure of mitochondrial mass^25^. In the pooled colon samples from right and left colon *MitoVolumeTotal* was higher in PD patients than controls (Fig. 3F; P = 0.017).

While there was no difference in mean mitochondrial volume (Fig. 2; *MitoVolumeMean*) between PD patients and controls (Fig. 3E; P = 0.197), these volumes showed a significant gradient from left to right colon in PD patients, but not in controls (Fig. 3G; P = 0.028 and Fig. 3H; P = 0.076). Taken together, PD patients displayed a lower *MitoVolumeMean* in the right colon than in the left colon, whereas controls did not.

**Figure 4:**
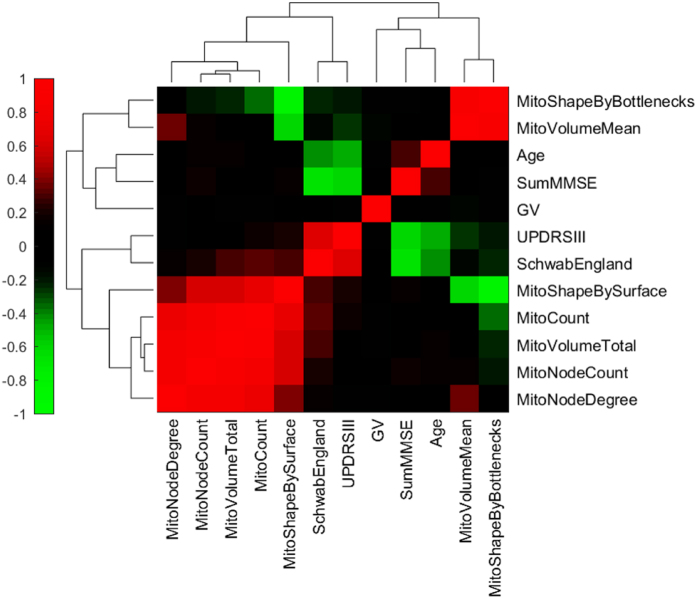
Analysis of correlation between clinical patient data, mitochondrial features, and ganglia volumes. Mitochondrial features are described in Fig. 2. GV, Ganglion volume; SumMMSE, Score from Mini-Mental State Examination; UPDRSIII, Motor part of the Unified Parkinson’s Disease Rating Scale; SchwabEngland, Score for individual levels of independence. Spearman’s rank correlation coefficients are color coded as indicated in the colorbar. Grouping of similar rows and columns in the matrix of coefficients, as illustrated by dendrograms, was done via hierarchical clustering with Euclidean distance metric and average linkage in Matlab.

We compared the mitochondrial features with the clinical parameters of PD patients. This revealed that decreasing mean mitochondrial volume was linked to an increasing UPDRS motor score, which reflects reduced motor performances (Fig. 4; P = 0.04; rho = −0.258, Spearman ranked test). Importanly, none of the features was linked to the age of the participants.

### Ganglion morphometrics

Average ganglion volume (Fig. 2; *GanglionVolume*) was significantly smaller in PD patients (17233 ± 14627 μm^3^) than in controls (27384 ± 22255 μm^3^) (Fig. 3I; P = 0.005). Comparing the right (n = 50) and the left colon (n = 56) of the whole cohort (including PD patients and controls), revealed that the *GanglionVolume* was larger in the right than in the left colon (Fig. 3J; P = 0.004). However, the *GanglionVolume* for the right colon was smaller in PD patients than in controls (Fig. 3K; P = 0.0006). A significant gradient in *GanglionVolume* along the colonic tract was observed in controls but not in patients (Fig. 3N,M). ROC curve analysis using the right-colon *GanglionVolume* revealed a high AUC value of 0.81, indicating that this feature displays high specificity and sensitivity in discriminating between PD patients and controls (Fig. 3L).

### Results extended by principal component analysis and support vector machine learning

**Figure 5:**
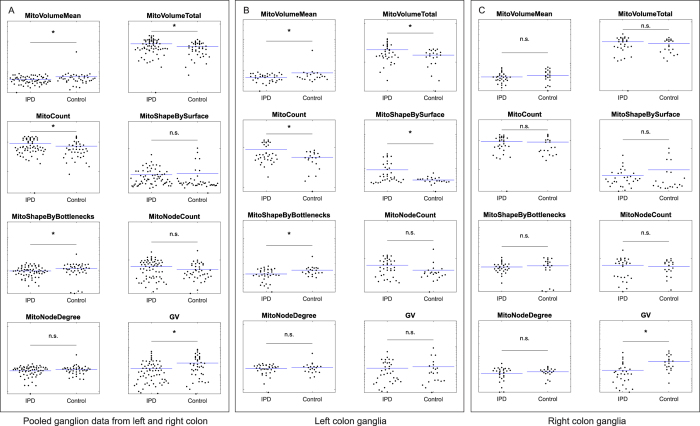
Ganglion and mitochondrial morphometric features. Comparision of pooled ganglion data from left and right colon between patients and controls (**A**). Comparison of left colon ganglia between patients and controls (**B**). Comparison of right colon ganglia between patients and controls (**C**). All features are plotted on log scale axes and described in Fig. 2.

**Figure 6:**
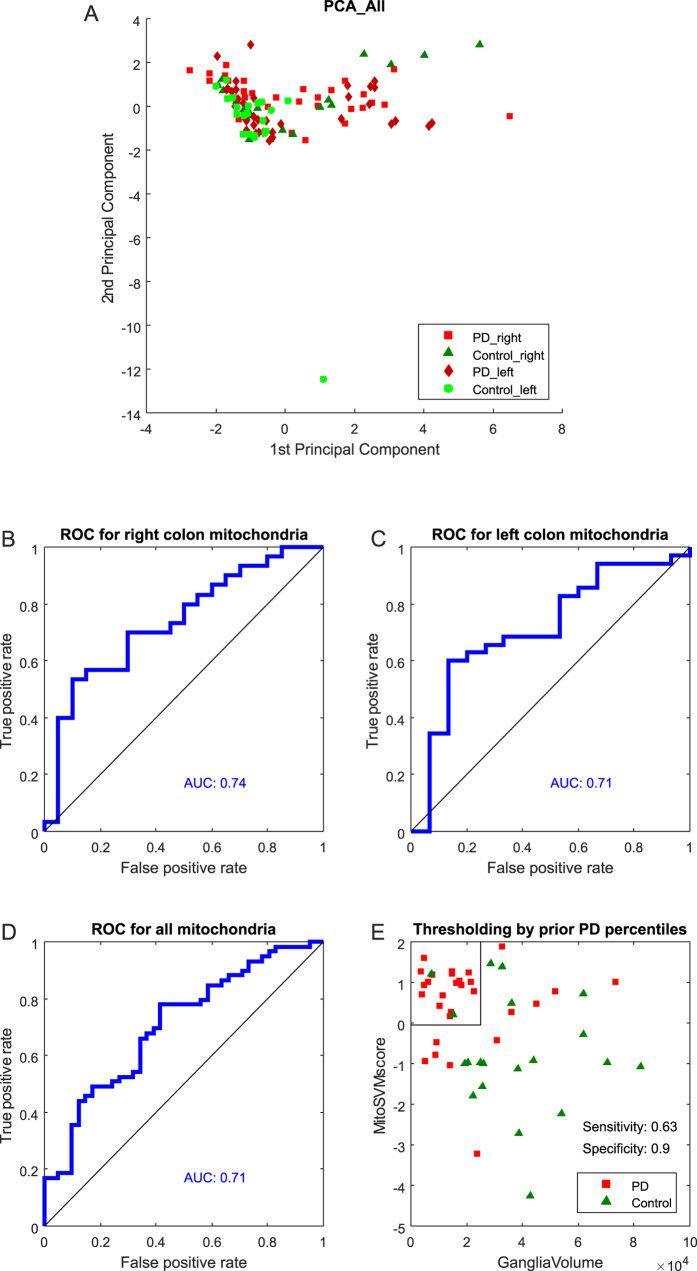
Evaluation of disease status classification. Principle component analysis of mitochondrial features in the whole study cohort (**A**). Each data point represents a single ganglion. Shapes and color codes indicate disease state and colonic sample location as shown in the legend. Evaluation of ganglion classification via mitochondrial morphometrics and support vector machines (**B**–**D**); AUC analysis was based on one hundred five-fold cross validations via support vector machine classification on patient or control ganglia. The combination of underlying mitochondrial scores and ganglion volumes in the right colon is used with the prior information that 60% of samples in this study are derived from patients and 40% from controls. Setting the MitoSVM score threshold to the quantile 0.6 of MitoSVM scores and the threshold for GangliaVolume - larger in patients than in controls - to the quantile 0.4 of GangliaVolumes provides a biomarker based classifier with a sensitivity of 0.63 and a specificity of 0.9 (**E**).

Feature-by-feature comparison for the pooled ganglion data revealed that five morphometric features differed between PD patients and controls (Fig. 5A). Also five features differed between PD patients and controls in the left colon (Fig. 5B). In contrast, a single feature differed between PD patients and controls in the right colon (Fig. 5C). Next, we performed a principal component analysis (PCA), that simultaneously considers all variables in a multidimensional space^26^,^27^,^28^. In the context of data visualization, PCA is widely used to reduce data dimensionality while retaining most of the variance of the data, thereby allowing graphic visualization^26^. It accomplishes this reduction by identifying directions, called Principal Components (PCs), along which the variance of the data is maximal. PCA revealed that a single outlier was present in the dataset (Fig. 6A; green circle). This information was used for the training of the SVM classifier, where parameters were set to assume 1% of outliers. Separate SVM analysis of the overall mitochondrial morphological phenotype in the right (AUC = 0.74) and in the left colon (AUC = 0.71), using the seven mitochondrial features, indicates that mitochondrial morphometrics provide useful information for the discrimination between PD patients and healthy control subjects (Fig. 6B–D).

### Integrating mitochondrial and ganglion morphometrics

Integrating mitochondrial *and* ganglion morphometric information might leverage the classification of PD patients and control subjects. To test this hypothesis we determined whether such an integration increases classification accurary. Right colon samples from patients displayed reduced ganglion volume (Fig. 5C) and an AUC-value with respect to mitochondrial morphometrics of 0.74 (Fig. 6B). When ganglion volume information was combined with information from mitochondrial morphometrics, two thresholds were required for classifying ganglia from PD patients or healthy subjects: one for the SVM score representing the predictive information from mitochondrial morphometrics, and one for ganglia volumes. These thresholds were set using the prior knowledge that 40% of the analyzed ganglia were derived from healthy controls and 60% from PD patients (Fig. 6E). The predictive power of a classifier combining both sets of information reached a sensitivity (true positive rate) of 0.63 and a specificity (1 - false positive rate) of 0.9 (Fig. 6E). This means that this classifier, independently from clinical diagnosis, could identify 63% of patients in this study as true positives. Importantly, 90% of the predicted patients would be correctly diagnosed by this combinatorial classifier while the remaining 10% would be false positives. At the same rate of 10% false positives, the sensitivity for ganglion volume based classification (Fig. 3L) and mitochondria based classification (Fig. 6D) was below 0.63 in the right colon. This reveals that combination of morphometric features from ganglia and mitochondria increases the classification accuracy of PD patients and healthy subjects.

## Discussion

Accumulating evidence suggests that aberrations in enteric neurons might be an early feature of PD^29^,^30^,^31^. To address this hypothesis we compared mitochondrial morphology between *ex vivo* enteric ganglia from PD patients (11 patients, 65 ganglia) and healthy age-matched control subjects (4 subjects, 41 ganglia).

### Morphometrics in 3D reveal mitochondrial changes in the enteric nervous system

Microdissection of colonic submucosa, introduced by Lebouvier *et al*.^9^,^32^ leveraged the immunofluorescence based study of ganglia. Here, we developed an image analysis algorithm for the quantification of mitochondrial morphometrics within ganglia volumes. To maximize spatial information retrieval and establish unbiased and reproducible morphometric measurements, the whole 3D image data set was analysed^33^,^34^.

Our data confirms that progressive mitochondrial fragmentation occurs in parallel with progressing motor deficits. An even more robust correlation with clinical findings may be foreseen in larger cohorts, with recruitment of patients in late stages of the disease, and with directly PD-linked gastrointestinal symptoms. The observed reduced ganglion volumes in PD patients are in line with the idea of enteric neurodegeneration as an early pathogenic event. Mitochondrial fragmentation, increased number of mitochondria, and increased total mitochondrial mass suggest a functional connection between mitochondrial morphofunction and ganglion degeneration.

### Rostrocaudal gradient of ganglion morphometrics in PD patients

The observed changes differed between the right and left colon of PD patients. Although PD patients did not display clinically apparent gastrointestinal dysfunction, their right colon ganglia had a reduced volume. Importantly, this right colon ganglion degeneration had predictive power to discriminate between PD patients and control subjects. The selective ganglion volume reduction in the right colon is in line with the previously proposed rostrocaudal gradient of enteric neuropathology along the digestive tube of PD patients^35^,^36^, characterized by a more pronounced pathology in proximal parts of the gastrointestinal tract, such as esophagus, than in distal parts of the gastrointestinal tract, such as the colon. We observed in this study that even within the colon such a gradient can be shown. Both healthy individuals and patients displayed a lower mean mitochondrial volume in the right colon relative to the left colon. This difference was significant for patients and borderline significant in the control group. To elucidate if mitochondrial changes are predominant in the right or left colon, each colon region was directly compared between patients and controls, as discussed in the next section.

### Lack of neuroprotective mitochondrial compensation in the right colon

Statistical comparison of single mitochondrial features between patients and healthy subjects only revealed significant differences in the left colon. Mitochondrial changes in the right colon could only be demonstrated using machine learning techniques that combined multiple features. This might suggest that significant mitochondrial structural changes in the right colon are absent or have already occurred at an earlier timepoint. Mitochondrial fragmentation has been proposed as one of the earliest subcellular markers of neurodegeneration^37^. This is in line with the observed mitochondrial fragmentation in the present cohort composed of PD patients at a rather early disease stage. However, the findings should be confirmed in PD patients at a yet earlier motor stage or in subjects with REM sleep behavior syndrome, an established forerunner syndrome of PD^38^. The increase in total mitochondrial mass in the left colon of PD patients might reflect a compensatory mechanism to sustain mitochondrial functioning^39^,^40^. Alternatively, increased mitochondrial mass might be due to impaired mitophagy^41^,^42^.The normal ganglion volumes observed in the left colon of PD patients support the idea of successful mitochondrial adaptation. Indeed, in fibroblasts derived from patients with Parkin mutation, paraquat exposure increased the mitochondrial mass but directly reduced mitochondrial branching and ATP production^43^.

The finding of intact ganglia in the left colon and shrunken ganglia in the right colon suggests a two-stage process: at first mitochondrial changes are efficiently compensatory and neuroprotective, but they are inexorably followed by the breakdown of cellular homeostasis further triggering cellular apoptosis and ganglia shrinking. Such a pathophysiological cascade is in line with the presented rostrocaudal gradient in mitochondrial fragmentation in PD. The rostrocaudal gradient theory is also supported by the fact that microbiota composition varies along the digestive tract^44^. A radial gradient in oxygene pressure from the intestinal tissue interface to the intestinal lumen indicates oxygene diffusion from host to microbiota^45^, which might interfere with oxidative phosphorylation in the enteric submucosa. Indeed, it is recognized that the brain-gut axis including central, autonomic, and enteric nervous systems is significantly modulated by gut microbiota^46^.

### Potential links between morphometric changes, alpha-synuclein, and pathogenesis

In complement to morphological changes in ganglion mitochondria, reported in the present study, prior studies reported abnormal protein aggregates in the colonic neurons of early-stage PD patients^7^,^8^,^9^,^10^,^11^. It has been proposed that misfolded alpha-synuclein directly alters mitochondria and their associated membranes^47^,^48^. In this perspective, the observed mitochondrial morphology changes are also in line with established neuropathological involvement of the enteric nervous system in PD^29^,^30^,^31^.

Due to lack of material, we did not evaluate the role of LB burden, so far considered to be primordial^7^,^8^,^9^. Although not uncontested, it had been proposed that ganglionic cell degeneration may be secondary to LB burden. Esophageal LB were first identified in PD patients with dysphagia^49^. Later LB were found in ganglia cells from the colonic myenteric plexus in a PD patient suffering from megacolon^29^. The presence of LB in other parts of the gastrointestinal tract was confirmed by others ever since^30^,^32^. Clinically asymptomatic patients with Braak stage 2 brain pathology also show LB pathology in ganglia located in the intramural and submucosa layers^50^. However, none of these studies analyzed mitochondrial alterations^51^,^52^. To the best of our knowledge, the present study is the first *ex vivo* study exploring mitochondrial morphometrics in the colon of PD patients.

The methodological approach is innovative for the field, but, importantly, requires deeper biopsy samples than usually collected. The thereby increased risk for injury and the true positive rate of only 63% vetoes the use of this method for large scale diagnostic screening. However, as colonoscopy is, at least in some countries, systematically used for colon cancer screening, and as the methods that we present here provide low false positive rates, 3D ganglion and mitochondria morphometrics offer new opportunities for the early validation of preliminary PD diagnosis. Furthermore, the presented approach could leverage the study of mitochondrial morphometrics in other neurodegenerative diseases such as Alzheimers’s disease, and Huntington’s disease, which have also been linked to mitochondrial dysfunction^53^. While mitochondrial sizes are at the resolution limit of conventional microscopy, our sophisticated method using advanced confocal microscopy, deconvolution, and computational image analysis, successfully manages this barrier and yields reproducible quantification of mitochondrial morphometrics within the ganglia. However, confirmation in larger cohorts and by another imaging approach such as electron microscopy or indirect proof by metabolic studies are essential.

We are aware of the caveats and restrictions of such a pioneer study. Mitochondrial morphometrics give only a snapshot view on the complex and rapidly ongoing process of mitochondrial dynamics. Within ganglia the view is limited to the direct surroundings of the nucleus and does not extend beyond the ganglia limits, to distal parts of the axons, and most importantly to the synaptic level^54^. Furthermore, the enteric neurons at the submucosal level essentially modulate mucosal processes, while those at the myenteric level control the motor activity, i.e., are responsible for dysmotility syndromes including constipation as seen in PD^55^. Separate analysis of the different enteric neuron subpopulations, such as inhibitory motor neurons, ascending interneurons and excitatory longitudinal muscle motor neurons may further enhance the discriminative power^56^. Future studies should include patients with overt dysmotility syndrome and patients at later motor stages of disease.

## Conclusion

Studying mitochondrial morphology in enteric neurons of PD patients is appealing as there are numerous similarities between enteric neurons and striatal dopaminergic neurons: high complexity and branching, low level of myelination, and long axon size. In both cell types mitochondria have to travel a long distance from the cell body to the dendritic or synaptic processes. Mitochondrial dynamics have to be performant, both in terms of adequate organelle fusion/fission and transport to distant cellular locations. High adaptability mediating robustness against mitochondrial stress has thus to be presumed, already in the healthy condition. With this analogy between enteric and striatal neurons in mind, our study is the first *ex vivo* proof in humans on mitochondrial dysfunction in Parkinson’s disease by detecting altered mitochondrial morphometrics in the colon.

## Methods

### Recruitment procedure, tissue preparation, staining and fluorescence microscopy

Colonoscopy in Luxemburg is offered within a colon cancer-screening program to individuals between the age of 50 and 80. Here this procedure was carried out with consecutive, non-demented patients with idiopathic PD, according to the London Brain Bank criteria^57^. These PD patients displayed no clinically apparent gastrointestinal symptoms and their age was within the above range. Healthy, age-matched volunteers were recruited as controls. All participants had given informed written consent prior to participating in this study. The study, the detailed experimental protocols as well as the information sheet for the patients were authorized and approved by the National Research Ethics Committee of Luxembourg (decision 201107/03), according to the rules established by the International Conference on Harmonisation (ICH) and the Declaration of Helsinki. All subjects underwent a standardized examination program as previously described^58^. All biopsies were taken by the same experienced gastroenterologist (W.J.). For each individual, at least one biopsy was obtained from the proximal/right colon tract and at least one biopsy was taken from the distal/left colon tract. Biopsies were stretched and pinned flat using needles (FST#26002-10, Minutien Pins, FST) using established protocols^9^,^32^, as illustrated (Fig. 1B). Submucosa was mechanically dissected from the mucosal tissue using a stereomicroscope and watchmaker’s forceps. The submucosa samples were fixed in 4% w/v paraformaldehyde for 2 h at room temperature. After three subsequent washing steps (10 min each), the specimen was permeabilized for 1.5 h in Dulbecco’s Phosphate Buffered Saline (D8537, SigmaAldrich, MO, USA) containing 1% v/v Triton X-100 (T8787, SigmaAldrich, MO, USA) and 1% w/v bovine serum albumin (A2058, SigmaAldrich, MO, USA). After another short wash, it was incubated for 45 minutes in a blocking solution (dPBS + 5% v/v goat serum, S-1000, Vector laboratories, Inc., CA, USA). We applied primary antibodies including anti-neurofilament-L (C-term) rabbit monoclonal antibody (Cat. #04-1112, Millipore), and anti-mitochondria mouse monoclonal antibody (Cat. #MAB1273, Millipore). These were diluted in dPBS containing 1% v/v Triton X-100 + 1% w/v BSA and incubated for 12 hours at a temperature of 4 °C. Following incubation with the primary antibodies, samples were washed three times with dPBS (10 minutes each) and then incubated for three hours at room temperature with secondary antibodies: Alexa-555 goat anti-rabbit (Cat. #A21428, Invitrogen) and Alexa-488 goat anti-mouse (Cat. #A11001, Invitrogen), diluted 1:500 in dPBS. After three additional washing steps with dPBS, the submucosa was mounted on a microscope glass slide (Fluoroshield with DAPI, Sigma-Aldrich) and covered with a cover glass. Images were acquired with a confocal microscope (LSM 710, Zeiss, Germany). Each biopsy slide was visually inspected by the operator using a 20× air objective (Zeiss, Plan-Apochromat, NA = 0.8) to locate ganglia. After this step, the area containing ganglion material was scanned using an objective with a higher magnification (63×, oil, Zeiss, Plan-Apochromat, NA = 1.4), resulting in a 3D image stack. The pinhole was set to acquire 1.5 μm sections. The size of z-steps was set to 0.18 μm. DAPI was excited using the 405 nm laser line. Fluorescence between 410–483 nm was filtered using a 3-Channel Quasar Detection Unit and detected using a photomultiplier tube (PMT). Alexa-488 was excited using the 488 nm laser line and fluorescence was detected using a 500–550 bandpass filter and a gallium arsenide phosphide detector. Alexa-555 was excited with a 561 nm laser and fluorescence was detected using a 565–610 bandpass filter and a gallium arsenide phosphide detector. All lightpaths passed a 488/561 main beam splitter. For DAPI, an additional 405 beamsplitter placed between 405 laser, main beam splitter, and sample was used. The gallium arsenide phosphide detectors were placed behind a 545 long pass filter while the light reflected from this filter was targeting the DAPI detector. All used filters, beamsplitters, and detection units are from Zeiss. The detection mode was set to photon counting. The size of acquired images was adjusted to ganglion size. Alexa-488 fluorescence was acquired in a first scan. DAPI and Alexa-555 fluorescence were acquired simultaneously in a second scan.

### Image analysis

3D image stacks were imported into AutoQuant X (version X3.0.3 64-bit edition, Media Cybernetics, Rockville, MD, USA) and deconvolved using an adaptive point spread function (10 iterations). Deconvolved stacks were further processed in Imaris 8.1.2 (Bitplane, Switzerland). Iso-surfaces of ganglia were reconstructed by manually selecting regions of interest (ROIs), corresponding to the convex hull of cells containing neurofilaments, for each individual slice. To export the ROI to Matlab 2014a (Mathworks, MA, USA), 3D surfaces were converted to ganglion volume masks. The image analysis procedure for mitochondrial analysis in Matlab, as well as the underlying settings for image processing, are presented in detail in the Results section.

### Statistical analysis

All investigators (except statisticians) were blinded for the identity of the barcoded samples. In total, a number of 89 anonymized biopsies (48 from the right colon and 41 from the left colon) were analyzed. In these biopsies 329 ganglia were identified (175 from the right and 154 from the left colon), 106 of which were randomly chosen for analysis using the default pseudorandom number generator provided by the R package, which uses the Mersenne-Twister algorithm. Whenever possible, at least two ganglia from the right and left colon were included for each subject. Significance of the differences between features were evaluated using a permutation test^59^: For comparing vectors of features, the true absolute mean difference was calculated. Then, the labels between compared vectors were randomly shuffled and the absolute mean difference was calculated. The random shuffling was repeated 100000 times. The P-value was calculated by comparing the true absolute mean with the absolute means from random shuffling. The classic approach to evaluate the diagnostic performance of a potential biomarker(s) is the receiver operating characteristic (ROC) analysis^60^,^61^, which plots the true-positive rate (sensitivity) against the false-positive rate (1-specificity) for a given biomarker or a combination of biomarkers. We applied a ROC analysis to determine whether mitochondrial and/or ganglial features could be used for discriminating between PD patients and healthy subjects. The area under the ROC curve (AUC) is a measure of the diagnostic performance (1 indicating a perfect diagnostic power while 0.5 indicates a futile biomarker). In addition to the above approaches, we used principal component analysis (PCA) for the data visualization, and linear support vector machines (SVM)^62^ for binary classification of PD patients and healthy subjects. To this end the SVM model was first trained using the whole dataset. The predictions obtained were then validated by running 100 experiments of 5-fold cross-validation using a linear SVM classifier. In this approach, each iteration uses 4/5 of the dataset to train the SVM classifier that determines how to split between classes (i.e., PD patients and healthy subjects), and then evaluates the rates of true and false positives in the remaining dataset. This way we ensure the independence between the training and test set, essential to ensure the generalization of the approach. Both PCA and SVM analysis were done in Matlab.

## Additional Information

**How to cite this article**: Baumuratov, A. S. *et al*. Enteric neurons from Parkinson’s disease patients display *ex vivo* aberrations in mitochondrial structure. *Sci. Rep.* **6**, 33117; doi: 10.1038/srep33117 (2016).

## Supplementary Material

Suplementary Figure 1

Supplementary video file

Supplementary movie legend
