# Supplementary material for: Enteric neurons from Parkinson’s disease patients display ex vivo aberrations in mitochondrial structure
Source: Sci Rep. 2016 Sep 14;6:33117. doi: 10.1038/srep33117 (PMC5021970; doi:10.1038/srep33117)
Supplement: Suplementary Figure 1 [file srep33117-s1.doc]

**Supplementary Figure 1**

**Enteric neurons from Parkinson’s disease patients**

**display *ex vivo* aberrations in mitochondrial structure.**

Baumuratov AS, Antony PMA, Ostaszewski M, He F, Salamanca L Antunes L, Weber J, Longhino L, Derkinderen P, Koopman WJH, Diederich NJ


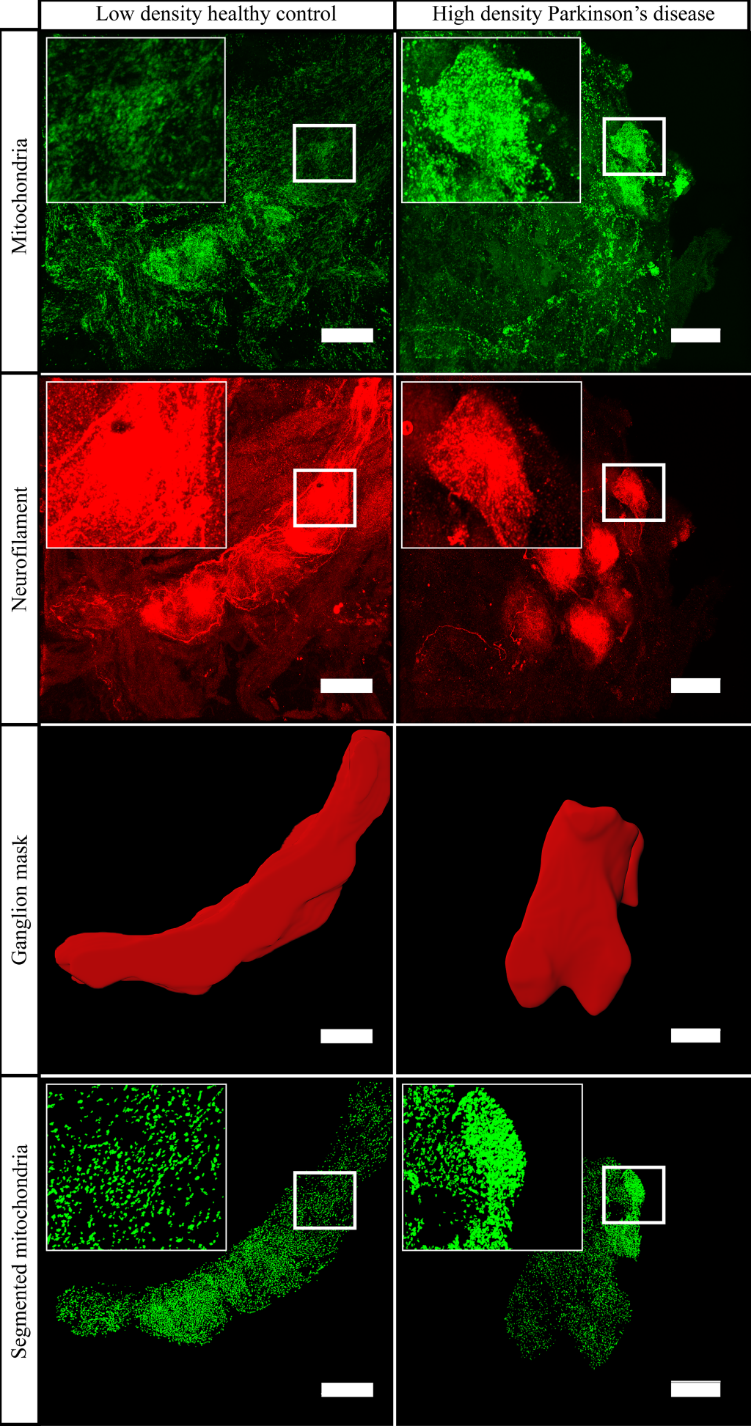


**Supplementary figure 1:** Examples of PD patient and control samples. The rows labeled ‘Mitochondria’ and ‘Neurofilament’ show deconvolved images. Ganglion masks were defined via manual selection from the neurofilament channel. Within the ganglium volume, the mitochondrial channel was analyzed via automated image analysis to segment mitochondrial volumes. The left part of the panel shows a ganglion from a control subject with low mitochondrial density. The right part shows a ganglion from a patient with Parkinson’s disease with high mitochondrial density. Scale bar = 20 µm. 3x magnified regions are highlighted with white boxes.
