# Supplementary material for: Enteric neurons from Parkinson’s disease patients display ex vivo aberrations in mitochondrial structure
Source: Sci Rep. 2016 Sep 14;6:33117. doi: 10.1038/srep33117 (PMC5021970; doi:10.1038/srep33117)
Supplement: Supplementary movie legend [file srep33117-s3.pdf]

Enteric neurons from Parkinson's disease patients display ex vivo aberrations in mitochondrial structure.

Baumuratov AS, Antony PMA, Ostaszewski M, He F, Salamanca L Antunes L, Weber J, Longhino L, Derkinderen P, Koopman WJH, Diederich NJ

Supplementary movie 1: Ganglion image analysis. Mitochondria and ganglion surfaces, eroded bodies, and skeletons in 3D
